# Metagenomic Next-Generation Sequencing Reveals the Profile of Viral Infections in Kidney Transplant Recipients During the COVID-19 Pandemic

**DOI:** 10.3389/fpubh.2022.888064

**Published:** 2022-07-11

**Authors:** Xiangyong Tian, Wenjing Duan, Xiulei Zhang, Xiaoqiang Wu, Chan Zhang, Zhiwei Wang, Guanghui Cao, Yue Gu, Fengmin Shao, Tianzhong Yan

**Affiliations:** ^1^Department of Urology, Henan Provincial Clinical Research Center for Kidney Disease, Henan Provincial People's Hospital, Zhengzhou University People's Hospital, Henan University People's Hospital, Zhengzhou, China; ^2^Department of the Clinical Research Center, Henan Provincial People's Hospital, Zhengzhou University People's Hospital, Henan University People's Hospital, Zhengzhou, China; ^3^Microbiology Laboratory, Henan Provincial People's Hospital, Zhengzhou University People's Hospital, Henan University People's Hospital, Zhengzhou, China; ^4^Department of Nephrology, Henan Provincial People's Hospital, Henan Provincial Key Laboratory of Kidney Disease and Immunology, Henan Provincial Clinical Research Center for Kidney Disease, Zhengzhou University People's Hospital, Henan University People's Hospital, Zhengzhou, China

**Keywords:** metagenomic next-generation sequencing, kidney transplantation, infection, virus, *torque teno virus*

## Abstract

**Background:**

To study the clinical application of metagenomic next-generation sequencing (mNGS) in the detection of viral infections in kidney transplant recipients (KTRs) during the COVID-19 pandemic.

**Methods:**

Using mNGS technology, 50 human fluid samples of KTRs were detected, including 20 bronchoalveolar lavage fluid (BALF) samples, 21 urine samples and 9 blood samples. The detected nucleic acid sequences were compared and analyzed with the existing viral nucleic acid sequences in the database, and the virus infection spectrum of KTRs was drawn.

**Results:**

The viral nucleic acids of 15 types of viruses were detected in 96.00% (48/50) of the samples, of which 11 types of viruses were in BALF (95.00%, 19/20), and the dominant viruses were *torque teno virus* (TTV) (65.00%; 13/20), *cytomegalovirus* (CMV) (45.00%; 9/20) and *human alphaherpesvirus 1* (25.00%; 5/20). 12 viruses (95.24%, 20/21) were detected in the urine, and the dominant viruses were TTV (52.38%; 11/21), *JC polyomavirus* (52.38%; 11/21), *BK polyomavirus* (42.86%; 9/21), CMV (33.33%; 7/21) and *human betaherpesvirus 6B* (28.57%; 6/21). 7 viruses were detected in the blood (100.00%, 9/9), and the dominant virus was TTV (100.00%; 9/9). Four rare viruses were detected in BALF and urine, including *WU polyomavirus, primate bocaparvovirus 1, simian virus 12*, and *volepox virus*. Further analysis showed that TTV infection with high reads indicated a higher risk of acute rejection (*P* < 0.05).

**Conclusions:**

mNGS detection reveals the rich virus spectrum of infected KTRs, and improves the detection rate of rare viruses. TTV may be a new biomarker for predicting rejection.

## Introduction

COVID-19 has rapidly escalated into a global pandemic, with more than 276.4 million cumulative cases and five million deaths worldwide as of December 2021 (1–3). The threats of the constantly mutating coronavirus continue to emerge. Variant strains with higher morbidity, stronger transmissibility, broader epidemic potential and higher mortality have been identified with the help of the development of genetic sequencing technology over the past 2 years (4–6). Although the worldwide epidemic of COVID-19 has imposed great challenges and heavy burdens on global public health, the work of transplantation clinicians has never halted (7, 8). That is because organ transplantation offers the greatest hope of survival and functional recovery for patients with irreversible end-stage organ failure. As far as the end-stage kidney disease patients are concerned, renal transplantation is the optimal treatment. And with the widespread application of potent immunosuppressive agents and the improvement of organ preservation techniques, the one-year survival rate of kidney transplantation has increased to more than 90% (9). However, with the long-term use of large amounts of immunosuppressants, the immune function of kidney transplant recipients (KTRs) is obviously impaired, increasing the chance of postoperative infection. Therefore, KTRs represent a population with an increased risk for COVID-19 and other pathogens infection, especially occult viral infections such as BK polyomavirus and cytomegalovirus, in which the outcomes of the infections are worse and in severe cases the infections may lead to graft loss and even death (10, 11).

KTRs can be simultaneously infected by multiple viruses. And their symptoms induced by infections sometimes are difficult to differentiate from rejections and drug application. Viral infections can't be identified by routine cultures. While detection of the viral genome by polymerase chain reaction (PCR) has several limitations, including difficulties in identifying multiple infections and low-throughput: only one species can be detected at a time, which causes the challenges for clinical treatment strategies and prognosis. Therefore, rapid determination of the presence of viral infections and effective improvement of accuracy and detection rates are urgent needs in the field of transplant infections.

Metagenome next-generation sequencing (mNGS) is an emerging method for pathogen identification. Since its successful use in the detection of new pathogenic infections in 2008, mNGS has gradually realized the transition from laboratory to clinical applications (12, 13). This culture-independent technique allows for rapid and accurate sequence detection of pathogenic microorganisms (including bacteria, fungi, viruses, and parasites) without bias by directly targeting nucleic acids in clinical samples. mNGS showed promising values in the rapid diagnosis of clinical infections, and can be applied in transplantation (14).

During the COVID-19 pandemic, factors of occult viral infections in KTRs are likely to be influenced by the COVID-19 epidemic in different ways and the management is more complicated, especially by telemedicine. However, few studies on this topic are currently available. Given this global background, in order to have a more definitive and comprehensive understanding of the viral pathogens following renal transplantation, we used mNGS in this study to identify virus spectrum of KTRs with symptoms from infection during this special period, hoping to provide a basis for improving clinical management strategies.

## Materials and Methods

### Study Design and Population

This study was performed in Henan Provincial People's Hospital, a tertiary teaching hospital in Zhengzhou, China. KTRs hospitalized with clinical symptoms and relevant signs of infection or unexplained fever, from May 2020 to May 2021, were enrolled. After recording demographic and clinical details, multifarious body fluid samples depending upon the site of infection at different stages were collected from enrolled KTRs, which were tested for viral infection profile by mNGS. Additional data on treatment, response to treatment, outcomes, and any relevant follow-up data were also collected. The results were reviewed by at least two clinicians to discriminate infection from colonization and contamination.

This study was approved by the Ethics Committee of the Henan Provincial People's Hospital [(2021)213], and all data were anonymised prior to analysis. The study was conducted in compliance with the Declaration of Helsinki.

### Sample Collection

The corresponding specimens were collected from each KTR enrolled in the study according to their symptoms. The exclusion criteria were consistent for all patients, namely: (1) patients with a previous history of multiple organ transplantation; (2) patients with samples sent for testing who were clearly considered contaminated; (3) kidney transplant patients with positive pregnancy tests. KTRs, who presented the most common pulmonary infection manifestations, including fever, cough, expectoration, shortness of breath, chest tightness, dyspnoea etc., were performed with chest CT for confirmation, and then the bronchoalveolar lavage fluids (BALF) samples collected during fiberoptic bronchoscopy were sent to laboratory for mNGS. Urinary tract infection is the second most common infection among KTRs, mainly manifests as lower urinary tract symptoms, including urinary frequency, urgency, pain, and a burning sensation during urination, with or without fever. KTRs with urinary tract infection were confirmed by routine urine test and quantitative urine culture, and then the clean midstream urine samples were sent to laboratory for mNGS. Peripheral blood samples were collected for mNGS from KTRs with fever of unknown origin or both of the above-mentioned infections. A 5-mL sample was taken per BALF or peripheral blood specimen. A 50-mL sample was taken per clean midstream urine specimen. All samples were collected according to standard operating procedures in accordance with the rules of aseptic technique, and were transported to the sequencing laboratory by cold chain in time.

### Metagenomic Next-Generation Sequencing and Data Analysis

Nucleic Acid Extraction: TIANamp Micro DNA Kit (DP316, TIANGEN BIOTECH, Beijing, China) was employed for the process of DNA extraction. Nucleic acid extraction were performed according to the manufacturer's operational guidebooks. DNA extraction was conducted for all samples.

Library Construction and Sequencing: The total DNA or cDNA was subjected to library construction through an end-repair method. A specific tag sequence was introduced at the end of each library. The library concentration was determined by Qubit 4.0 nucleic acid fluorescence quantitative analyzer (Q33226, Thermo Fisher, USA) and Qubit® dsDNA HS Assay Kit (Q32854, Thermo Fisher, USA). Agilent 2100 Bioanalyzer (G2939BA, Agilent, USA) was used to evaluate the DNA concentration and fragment size in the library to be sequenced for the quality control of the DNA libraries. DNA nanospheres were prepared by one-step DNB Kit (1000025076, Huada Zhizao, China). Quality qualified libraries were sequenced by MGISEQ-200 platform.

Bioinformatic Analysis: After removing low-quality (< 35 bp) and low-complexity reads according to prinseq (version 0.20.4), and computational subtracting the human host sequences mapped to the human reference genome (hg38) from the sequencing data by Burrows-Wheeler Alignment (0.7.10-r789), high-quality sequences were generated (15). The remaining non-host sequences were matched and classified with dedicated viral databases which were downloaded from the National Center for Biotechnology Information (ftp://ftp.ncbi.nlm.nih.gov/genomes/) and other public databases. So far, more than 4,000 viral genomes were contained in the integrated classification reference databases. The mapped data were processed for advanced data analysis. Lists of suspected pathogenic viruses were produced, which included the numbers of strictly mapped reads, coverage rate, and depth (16). The clinical diagnosis was determined by considering all the clinical manifestations, possible pathogens identified by mNGS and other laboratory tests together.

### Statistical Analysis

Continuous data were expressed as the mean ± standard deviation (SD). Counting data were expressed as the number of cases with percentage (%). The Chi-square test was conducted for comparing the rate of low concentration group and high concentration group, rejection group and non-rejection group. Data analyses were performed using Statistical Package for the Social Sciences (SPSS) version 24.0 statistical software (IBM SPSS, Chicago, IL, USA) (17). All *P*-values were two-sided, and statistical significance was defined as *P* < 0.05.

## Results

### Characteristics of the Patients

A total of 39 KTRs were investigated in the present study. A total of 50 samples were collected from these KTRs at different infection phases, including 20 BALF samples, 21 urine samples and 9 blood samples. Abstractions of patients' demographic and clinical information were collected, including age, sex, dialysis durations, comorbidity, infection signs, application of immunosuppression, laboratory examinations, as summarized in Tables 1, 2. The median age of all KTRs was 39.49 (range 20–58) years. The clinical signs on physical examination of the KTRs were heterogeneous and non-specific. Mean values of lymphocyte count and lymphocyte proportion were both below the normal range.

**Table 1:** Baseline characteristics of participants.

**Characteristics**	**Cases (*n =* 39)**
Gender	
Male	22 (56.41%)
Female	17 (43.59%)
Age (years)	39.49 ±10.42
Pre-transplant dialysis durations (months)	25.51 ± 32.92
Comorbidity	
Hypertension	32 (82.05%)
Hypertension and Diabetes	1 (2.56%)
Immunosuppressant	
Tac+MMF+Pred	32 (82.05%)
Tac+MPS+Pred	7 (17.95%)

*Data were provided as n / percentage (%) or mean ± standard deviation. Tac, tacrolimus; MMF, mycophenolate mofetil; MPS, mycophenolate sodium; Pred, prednisone*.

**Table 2:** Baseline characteristics of patients.

**Characteristics**	**Samples (*n =* 50)**
Body temperature	
Normal	21 (42.00%)
≥37.3°C	29 (58.00%)
Serum creatinine (umol/L)	148.26 ± 84.22
Lymphocyte count (x10^9^/L)	0.80 ± 0.57
Lymphocyte ratio (%)	14.64 ±12.09
Acute rejection	
Yes	17 (34.00%)
No	33 (66.00%)
Tacrolimus blood concentration	
≥8 ng/ml	13 (26.00%)
<8 ng/ml	37 (74.00%)

*Data were provided as n / percentage (%) or mean ± standard deviation. Normal lymphocyte count: (1.1–3.2) × 10^9^/L; Normal lymphocyte ratio: 20–50%*.

The same renal transplant team performed all the surgical procedures and postoperative management together. Routine standard of care and post-transplant medication including immunosuppressive drug therapy was administered in accordance to center standard. The standard immunosuppressive protocol included induction with anti-thymocyte globulin (ATG), followed by maintenance immunosuppressive regimen consisting of tacrolimus, mycophenolate mofetil (MMF) / mycophenolate sodium (MPS) and prednisone. The dosage of tacrolimus was weight-based (0.05 mg/kg twice daily) started at the time of transplantation and then adjusted according to close monitoring to maintain tacrolimus blood concentrations within the therapeutic range (6–8 ng/ml) to ensure efficacy and safety.

### Diagnostic Performance of mNGS in Three Sample Types

Viral nucleic acids were detectable in 48 of 50 samples (96.00%), for a total of 15 virus types. Of these, 19 (95.00%) of the 20 BALF samples were positive for viral nucleic acids, for a total of 11 virus types, of which the top five were torque teno virus (TTV) in 13 cases (65.00%), human betaherpesvirus 5 (Cytomegalovirus, CMV) in 9 cases (45.00%), human alphaherpesvirus 1 in 5 cases (25.00%), human gammaherpesvirus 4 (Epstein-Barr virus, EBV) in 3 cases (15.00%), and human betaherpesvirus 7 in 2 cases (10.00%). Mixed viral infections were observed in 11 cases (55.00%).

Among the 21 urine samples, viral nucleic acids were detected in 20 cases (95.24%) with 12 virus types, of which the top five were TTV in 11 cases (52.38%), JC polyomavirus (JCV) in 11 cases (52.38%), BK polyomavirus (BKV) in 9 cases (42.86%), CMV in 7 cases (33.33%), and human betaherpesvirus 6B in 6 cases (28.57%). Mixed viral infections were observed in 18 cases (85.71%).

**Figure 1:**
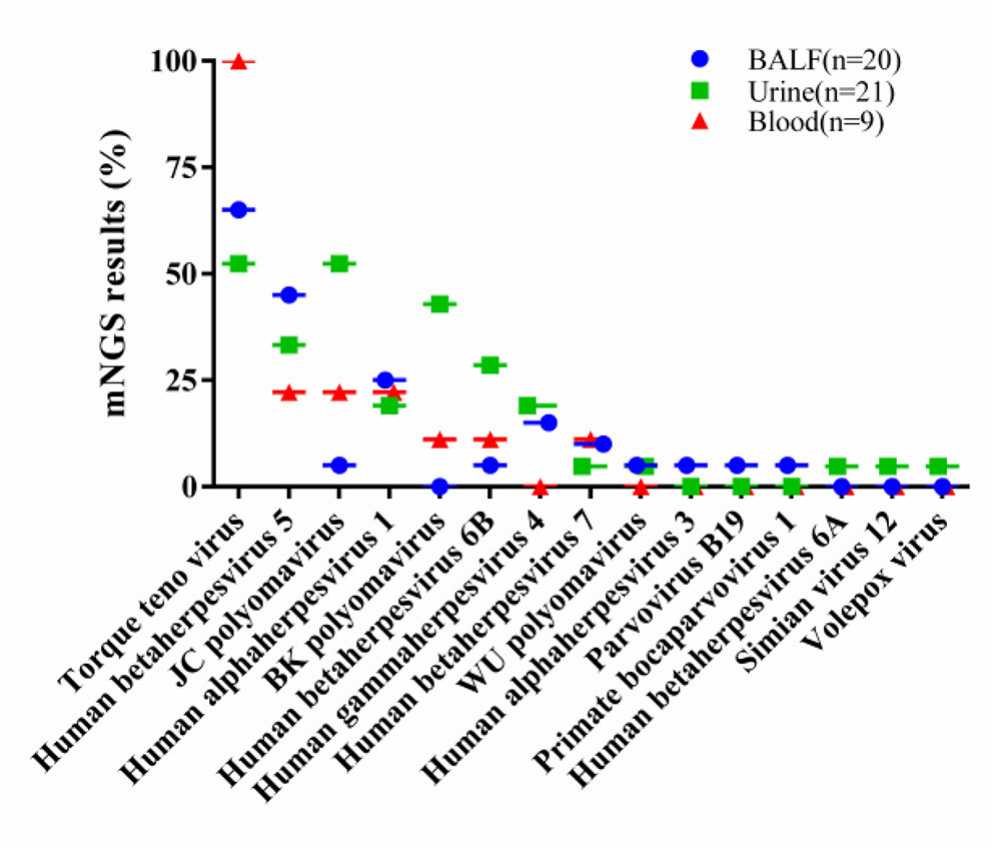
The percentage of each virus in their respective samples, including 20 BALF samples, 21 urine samples and 9 blood samples. BALF, bronchoalveolar lavage fluids.

Viral nucleic acids were detected in all 9 samples of peripheral blood (100.00%) with 7 virus types, namely, 9 cases of TTV (100.00%), 2 cases each of human alphaherpesvirus 1, CMV and JCV (22.22%), 1 case each of BKV, human betaherpesvirus 6B and human betaherpesvirus 7 (11.11%). Four cases were mixed viral infections (44.44%) (Figure 1).

### Relative Abundance of Viral Nucleic Acids in Three Types of Samples

**Figure 2:**
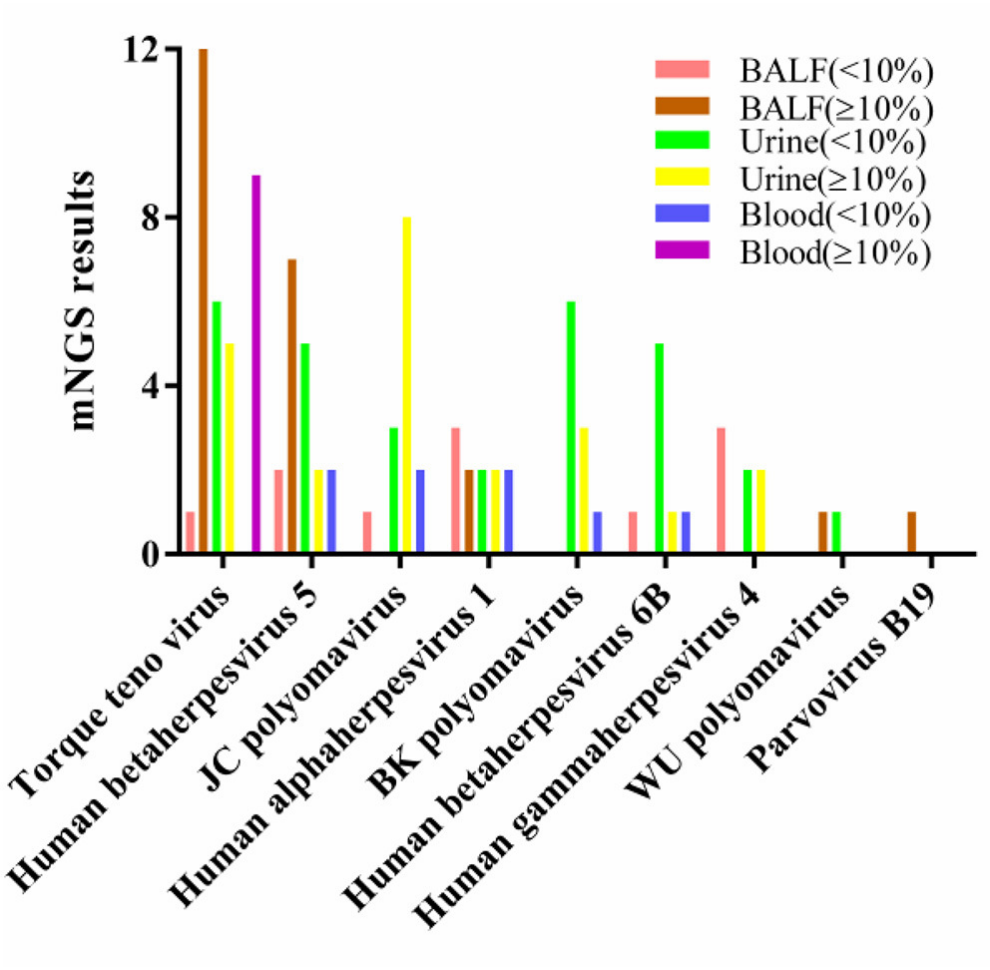
Relative abundance of viral nucleic acids in three types of samples. BALF, bronchoalveolar lavage fluids.

We used 10% as the threshold to screen out virus types with high relative abundance, and the results showed that there were 5 virus types in the alveolar lavage fluid, namely, TTV(*n* = 12), CMV (*n* = 7), human alphaherpesvirus 1 (*n* = 2), WU polyomavirus (*n* = 1), and parvovirus B19 (*n* = 1). Urine had 8 virus types, namely, JCV (*n* = 8), TTV (*n* = 5), BKV (*n* = 3), CMV (*n* = 2), human alphaherpesvirus 1 (*n* = 2), EBV (*n* = 2), human betaherpesvirus 6A (*n* = 1) and human betaherpesvirus 6B (*n* = 1). There was only TTV in peripheral blood (*n* = 9) (Table 3). The only virus whose relative abundance exceeded 10% in all three samples was TTV (Figure 2).

**Table 3:** Relationship between tacrolimus concentration and viral infection.

	**LCG**	**HCG**	**χ^2^-value**	***P*** **-value**
All cases	37	13		
Reads≥10	25 (67.57%)	11 (84.62%)	0.670	0.413
Reads≥100	13 (35.14%)	7 (53.85%)	1.403	0.236
TTV positive cases	22 (59.46%)	12 (92.31%)	3.380	0.066
Reads≥10	9 (24.32%)	5 (38.46%)	0.954	0.329
Reads≥100	6 (16.22%)	4 (30.77%)	0.526	0.468

*Data were provided as n / percentage (%). LCG, low concentration group; HCG, high concentration group; TTV, torque teno virus*.

### Reads of Viral Nucleic Acids Detected in Three Types of Samples

In BALF, 14 cases (70.00%) had a total viral nucleic acid reads ≥10 and 6 cases (30.00%) had nucleic acid reads ≥100, The viruses with reads ≥100 were TTV (*n* = 4), CMV (*n* = 2), and *WU polyomavirus* (*n* = 1).

In urine, 14 cases (66.67%) had a total viral nucleic acid reads ≥10, 10 cases (47.62%) had nucleic acid reads ≥100. The viruses with reads ≥100 were JCV (*n* = 6), CMV (*n* = 3), TTV (*n* = 2), BKV (*n* = 1), *human alphaherpesvirus 1* (*n* = 1), *human betaherpesvirus type 6A* (*n* = 1), and *simian virus 12* (*n* = 1).

In peripheral blood, 8 cases (88.89%) had a total viral nucleic acid reads ≥10 and 4 cases (44.44%) had nucleic acid reads ≥100, which were TTV (*n* = 4).

**Figure 3:**
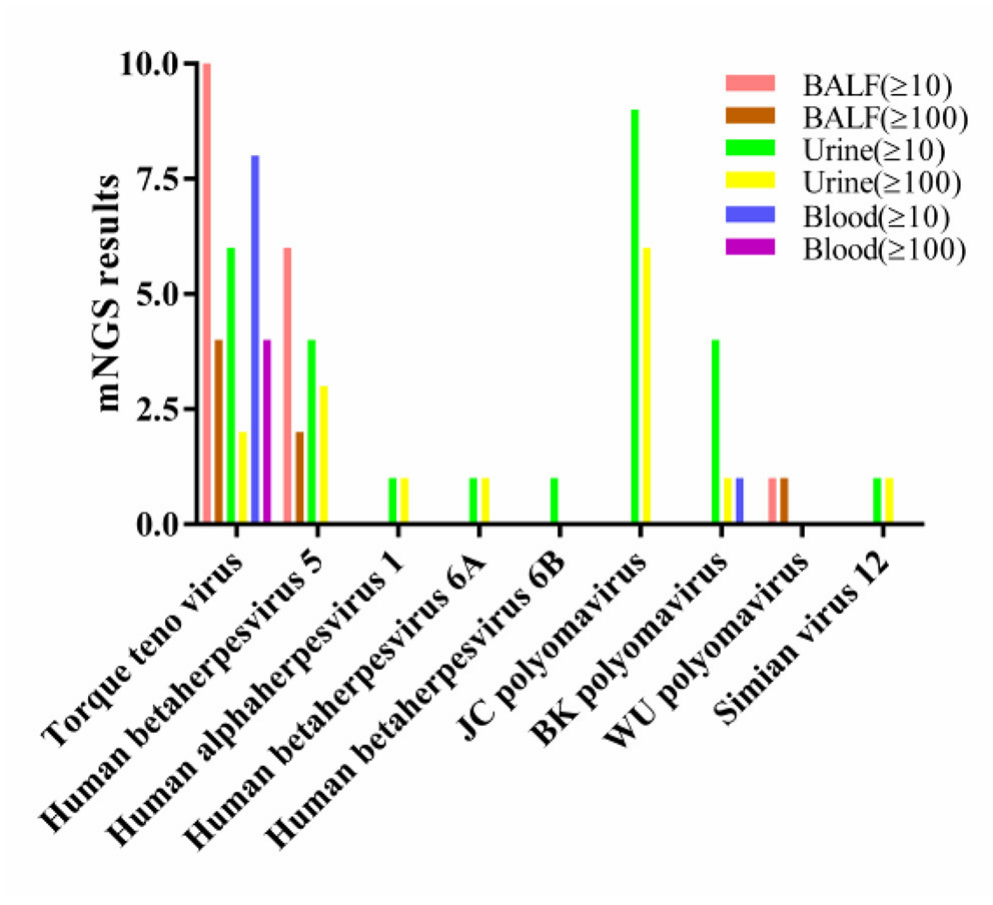
Viral nucleic acid number of detected reads in three types of samples. BALF, bronchoalveolar lavage fluids.

The only virus with a viral nucleic acid reads of more than 100 in all three samples was TTV (Figure 3).

### mNGS Improves the Detection of Rare Viruses

mNGS significantly improved the detection rate of rare or uncommon viruses. Four rare viruses were detected in this study: WU polyomavirus, simian virus 12, volepox virus and primate bocavirus type 1. Among them, simian virus 12 and volepox virus were detected once in urine samples. Primate baculovirus type 1 was detected once in alveolar lavage fluid. WU polyomavirus was detected in one KTR's alveolar lavage sample and another KTR's urine sample, respectively.

Nucleic acid reads were less than 10 for all viruses except for WU polyomavirus in one BALF sample and simian virus 12 in one urine sample, which had nucleic acid reads greater than 100.

### Relationship Between Tacrolimus Concentration and Viral Infection

We divided all samples into low concentration group (LCG, *n* = 37) and high concentration group (HCG, *n* = 13) according to whether the tacrolimus concentration was ≥8ng/ml, and found that there were 25 (67.57%) and 11 (84.62%) cases with total viral nucleic acid reads ≥10 in the LCG and the HCG, respectively. There were 13 (35.14%) and 7 (53.85%) cases with viral nucleic acid reads ≥100 in the two groups, respectively. No statistical differences were found between the two groups (*P* > 0.05).

We focused on the infection of the TTV in the LCG and HCG, and found that the nucleic acid of the virus was detected in 22 (59.46%) and 12 (92.31%) cases, respectively, with number of detected reads ≥10 in 9 (24.32%) and 5 (38.46%) cases, and ≥100 in 6 (16.22%) and 4 (30.77%) cases, respectively, without statistical difference between the two groups (*P* > 0.05) (Table 3).

### Relationship Between Viral Infection and Rejection After Renal Transplantation

We compared the viral infections in the rejection group (*n* = 17) and the non-rejection group (*n* = 33) and found that the total nucleic acid reads ≥10 was detected in 16 (94.12%) and 20 (60.61%) cases in the two groups, respectively, with statistically significant difference between the two groups (χ^2^ = 4.698, *P* = 0.030). The patients number of detected reads ≥100 was 10 cases (58.82%) and 10 cases (30.30%) in the two groups, respectively, and there was no statistically significant difference between the two groups (*P* > 0.05).

We compared the TTV infection in the rejection and non-rejection groups and found that TTV nucleic acid reads were detected in 14 (82.35%) and 20 (60.61%) cases, respectively, with no statistical difference between the two groups (*P* > 0.05). Reads ≥10 were detected in 12 (70.59%) and 11 (33.33%) cases, respectively, with statistical difference in comparison (χ^2^ = 6.269, *P* = 0.012). There were 8 (47.06%) and 2 (6.06%) cases with reads ≥100, respectively, with a statistical difference in comparison (χ^2^ = 9.364, *P* = 0.002) (Table 4).

**Table 4:** Relationship between viral infection and rejection after renal transplantation.

	**Rejection group**	**Non-rejection group**	**χ^2^-value**	***P*** **-value**
All cases	17	33		
Reads≥10	16 (94.12%)	20 (60.61%)	4.698	0.030
Reads≥100	10 (58.82%)	10 (30.30%)	3.803	0.051
TTV positive cases	14 (82.35%)	20 (60.61%)	1.542	0.214
Reads≥10	12 (70.59%)	11 (33.33%)	6.269	0.012
Reads≥100	8 (47.06%)	2 (6.06%)	9.364	0.002

*Data were provided as n / percentage (%). TTV, torque teno virus*.

### Treatments and Outcomes

All patients were given empirical anti-infective therapy upon admission, and the dose of immunosuppressive drugs was reduced after definitive infection, i.e., MMF or EC-MPS was reduced or discontinued directly, and tacrolimus was maintained in small doses or reduced appropriately. Ganciclovir or penciclovir was also administered. All patients were cured with anti-infective treatment and combination therapy.

Among those with pulmonary infections, one case resulted in eventual failure of the transplanted kidney due to infection and rejection, and the function of the transplanted kidney was affected in two patients due to infection and rejection. Among those with urinary tract infections, 8 cases had recurrent urinary tract infections the function of the transplanted kidney was affected in 1 case.

## Discussion

The current world is still going through a rough patch for the outbreak of COVID-19. It causes public health concerns especially in the field of transplantation. KTRs undergoing post-transplant immunosuppressive therapy are at the risk of infection (18). Although many transplant practitioners have studied one or more pathogenic infections in KTRs, studies revealing their viral profile have not been seen. Fortunately, mNGS technology, with the characteristics of fast detection speed, high sensitivity and wide coverage, is able to effectively compensate for the deficiency of traditional culture and PCR, particularly offer a very significant practical advantage for KTRs (19–21). Since traditional culture cannot detect viruses and PCR is low-throughput, the advantage of mNGS for virus detection is highlighted. It directly extracts all viral nucleic acid fragments in the specimen, compares the reference sequences in the specific database with the specimen sequences, analyzes them by intelligent algorithms to obtain viruses in the specimen that have the same sequences as various reference pathogens, avoiding the missed detection of difficult-to-identify viruses (22). The findings of this research shed new light onto the viral infection profile in KTRs during the COVID-19 pandemic.

mNGS has helped researchers and clinicians solve many intractable diseases since its initial clinical application. As tested by Miao et al., the sensitivity of mNGS (50.7%) was higher than traditional method (35.2%). They considered that mNGS could yield a higher sensitivity for pathogen identification and be less affected by prior antibiotic exposure, thereby emerging as a promising technology for detecting infectious diseases (20). Jerome et al. (23) performed mNGS among 40 febrile returning travelers for the pathogenic diagnosis, avoiding the missed detection by traditional methods, which indicated that mNGS had the potential to be an all-in-one rapid diagnostic testing. Palacios et al. (12) reported that 3 patients who received the same donor organ died of high fever within 4–6 weeks, but the results of traditional culture were negative. With the help of mNGS, the patients were clearly diagnosed as arenaviruses infection, which revealed the mystery of pathogenic microorganisms. Gazzani reported a case of fatal disseminated cowpox virus infection in an adolescent renal transplant recipient, which was greatly assisted by mNGS (24).

In our study, viral nucleic acids were detected in 96.00% of the samples, involving 15 virus types. Urine positivity rate was 95.24% with 12 virus types, and the predominant viruses were TTV, JCV, BKV, CMV, and human betaherpesvirus 6B. The virus-positive rate in urine samples was 95.24%, and a total of 12 viruses were detected, among which the predominant viruses were TTV, JCV, BKV, CMV, and human betaherpesvirus 6B. 95.00% BALF was positive for a total of 11 virus types, with the predominant viruses being TTV, CMV, human alphaherpesvirus 1, EBV, and human betaherpesvirus 7. Peripheral blood was 100.00% positive for 7 virus types, with the predominant virus being TTV. All peripheral blood samples were virus-positive, and TTV was the predominant one among the 7 viruses detected. It is estimated that 5–8% of KTRs are infected with BKV in the first 3 years after transplant, which can lead to nephropathy, impaired kidney function and graft loss (25–28). In this study, BKV fragments were detected in only 9 urine samples and 1 blood sample, and only 1 urine sample had a number of detected reads more than 100. Accordingly, it is hypothesized that the relatively low positive rate of BKV and viral load in infected KTRs is probably due to the reduction of immunosuppression. Herpesvirus infections, especially CMV infection, have also been well studied (27, 29, 30). The number of studies on JCV infection is also increasing (31, 32). We also detected TTV (33–35) and parvovirus B19 (36–38), which are still relatively rare in the field of transplantation. Four rare virus types, WU polyomavirus, primate bocavirus type 1, simian virus 12, and vole pox virus, have also been detected and have not been reported in KTRs before, so we cannot be sure if they caused the disease, but these patients eventually recovered with treatment. Therefore, mNGS significantly improved the detection rate of common and rare viruses in KTRs, and the application of mNGS in the renal transplant is worth promoting.

Next, we studied the nucleic acid read length and relative abundance of the viruses in each sample, and found that the top virus in BALF and blood was TTV, while in urine, the top virus was JCV, followed by TTV. Notably, only TTV was positive in all three types of samples with nucleic acid reads ≥100 and relative abundance ≥10%, which was completely unexpected. The detection rate of the TTV was 66.00% in all samples, and 100.00% in blood samples, which is consistent with the findings of previous studies (33, 34, 39). In addition to the detection in these types of samples, detection of the TTV in cerebrospinal fluid has also been reported (40), suggesting that the TTV may be widely present in human body fluids and may be particularly evident in immunosuppressed population. An Australian cross-sectional study noted that TTV was detectable in the plasma of 93% of KTRs, suggesting that TTV may be a novel marker for immunosuppressive therapy for KTRs (39). Another study has also identified TTV as a predictor of the level of immunosuppression and infection after solid organ transplantation (41, 42). We compared TTV infection in the LCG and HCG. Although the difference did not achieve statistical significance, we believe that this was due to the very small sample size. The TTV positivity rate was lower in the LCG than in the HCG (59.46 vs. 92.31%) and consistent results were obtained in cases with nucleic acid reads ≥10 and ≥100. It is suggested that the higher the tacrolimus concentration is, the more susceptible the KTRs are to be infected by the virus and higher the viral load is.

Further, we compared viral infections in the rejection and non-rejection groups, and the rates of viral infections with nucleic acid reads ≥10 were 94.12 and 60.61% in the two groups, respectively, which were statistically different (*P* < 0.05), suggesting that the rejection group is more likely to lead to viral infections. Specifically for TTV, we found that the infection rate was higher in the rejection group than in the non-rejection group (82.35 vs. 60.61%). Although the difference was not statistically significant in the primary analysis, the trend was obvious. Moreover, the stratified analysis of viral nucleic acid read lengths showed that the infected cases was significantly more in the rejection group than in the non-rejection group (70.59 vs. 33.33% for acid reads ≥10, and 47.06 vs. 6.06% for reads ≥100), indicating a higher proportion of KTRs with TTV load in the rejection group. These results suggested that TTV may be a potential biomarker for predicting renal transplant rejection, which was also tentatively validated by Strassl et al. (43).

Despite the great value of mNGS in infectious diseases, especially rare infectious diseases, there are still many practical problems in its clinical application. More than 99% of the reads generated by sample sequencing are from human hosts (44), while microorganisms represent only a small percentage. And sequencing all nucleic acids reduces the sensitivity of pathogen identification, making it difficult to distinguish between colonizing, background and pathogenic bacteria among the various species detected (45). But it is possible to deplete host nucleic acids by certain methods (46, 47), and reducing the human-derived nucleic acid sequence proportion can increase the amount of microbial data and improve sensitivity to some extent. In any case, the determination of mNGS results requires a combination of nucleic acid fragment counts, clinical presentation, other laboratory results and background microorganisms.

In conclusion, we revealed the viral profile of KTRs by mNGS technology. Certain viruses infection such as TTV may be a reflection of the degree of immunity in KTRs, as well as a potential biomarker for predicting rejection. Therefore, mNGS is recommend as a routine testing for KTRs to make real and lasting benefits for health and healthcare.

## Data Availability Statement

The datasets presented in this study can be found in online repositories. The names of the repository/repositories and accession number(s) can be found below: https://www.ebi.ac.uk/metagenomics~ERP136978.

## Ethics Statement

The studies involving human participants were reviewed and approved by Henan Provincial People's Hospital. The patients/participants provided their written informed consent to participate in this study. Written informed consent was obtained from the individual(s) for the publication of any potentially identifiable images or data included in this article.

## Author Contributions

XT and WD drafted the manuscript. XT and XZ carried out the statistical analysis. XW, CZ, ZW, GC, and YG interpreted data. XT, WD, FS, and TY participated in the design of the study and coordination. All authors contributed to the article and approved the submitted version.

## Funding

This work was supported by the Project of Science and Technology of Henan Province (Grant No. 202102310438), the 23456 Talent Project Foundation of Henan Provincial People's Hospital (Grant No. ZC23456127), Joint construction project of Henan Medical Science and technology research plan (Grant No. LHGJ20210042), and Foundation of Henan Educational Committee (Grant No. 22A320012).

## Conflict of Interest

The authors declare that the research was conducted in the absence of any commercial or financial relationships that could be construed as a potential conflict of interest.

## Publisher's Note

All claims expressed in this article are solely those of the authors and do not necessarily represent those of their affiliated organizations, or those of the publisher, the editors and the reviewers. Any product that may be evaluated in this article, or claim that may be made by its manufacturer, is not guaranteed or endorsed by the publisher.
